# Arterial compression of nerve is the primary cause of trigeminal neuralgia

**DOI:** 10.1007/s10072-013-1518-2

**Published:** 2013-08-21

**Authors:** Guo-qiang Chen, Xiao-song Wang, Lin Wang, Jia-ping Zheng

**Affiliations:** Department of Neurosurgery, Yuquan Hospital of Tsinghua University, 5 Shijing Shan Road, Shi Jing Shan District, Beijing, 100049 China

**Keywords:** Demyelination, Hemifacial spasm, Microsurgical decompression, Nerve compression, Trigeminal neuralgia

## Abstract

Whether arterial or venous compression or arachnoid adhesions are primarily responsible for compression of the trigeminal nerve in patients with trigeminal neuralgia is unclear. The aim of this study was to determine the causes of trigeminal nerve compression in patients with trigeminal neuralgia. The surgical findings in patients with trigeminal neuralgia who were treated by micro vascular decompression were compared to those in patients with hemifacial spasm without any signs or symptoms of trigeminal neuralgia who were treated with microvascular decompression. The study included 99 patients with trigeminal neuralgia (median age, 57 years) and 101 patients with hemifacial spasm (median age, 47 years). There were significant differences between the groups in the relationship of artery to nerve (*p* < 0.001) and the presence of arachnoid adhesions (*p* < 0.001) but no significant difference in relationship of vein to nerve. After adjustment for age, gender, and other factors, patients with vein compression of nerve or with artery compression of nerve were more likely to have trigeminal neuralgia (OR = 5.21 and 42.54, *p* = 0.026 and *p* < 0.001, respectively). Patients with arachnoid adhesions were less likely to have trigeminal neuralgia (OR = 0.15, *p* = 0.038). Arterial compression of the trigeminal nerve is the primary cause of trigeminal neuralgia and therefore, decompression of veins need not be a priority when performing microvascular dissection in patients with trigeminal neuralgia.

## Introduction

Trigeminal neuralgia is characterized by severe facial pain in the distribution of the trigeminal nerve that is paroxysmal, provokable, unilateral, and not accompanied by sensory loss [1, 2]. The pain typically lasts only seconds, but is described as excruciating, and is triggered by sensory stimuli that can include chewing, yawning, and speaking [3]. The pain is episodic and pain-free intervals can range from day to years. The incidence of trigeminal neuralgia is reported to be approximately 4 per 100,000 population and gradually increases with age; the condition is rare before 40 years of age and the average age of onset is 60 years [2–5].

Though the condition has been well-studied, there is still debate regarding the pathophysiology. Most experts agree that the etiology is segmental demyelination of trigeminal sensory nerves in the nerve root or brainstem, and the demyelination is due to chronic compression of the nerve root where it exits from the pons [3, 6, 7]. Compression can be due to vascular abnormalities such as an aneurysm or arteriovenous malformation [8], and demyelination due to multiple sclerosis has been described [6]. Most theories, however, consider compression from an artery and/or vein (e.g., superior cerebellar) as the trigeminal nerve exits the pons as the cause [3, 5, 9]. Though vascular compression is the most widely accepted theory, other theories such as bioresonance have been examined [10].

Medical treatment for trigeminal neuralgia primarily consist of anticonvulsants [11]. Carbamazepine is the first-line treatment, and other that are used include phenytoin, oxcarbazepine and lamotrigine. Other medications that can be tried include gabapentin and baclofen. When medical treatment is ineffective, or a patient cannot tolerate the side effects, the primary surgical treatment is microvascular decompression.

Microvascular decompression is performed based on the hypothesis that compression of the trigeminal nerve is responsible for the demyelination and pain [12–15]. The procedure is performed via a retrosigmoid posterior fossa craniotomy, and the root of the trigeminal nerve is explored for signs of compression, which are then relieved by microdissection and moving the vessel causing the compression. The most common vessels identified as causing compression are the superior cerebellar artery and anterior inferior cerebellar artery [12–15]. However, there is debate regarding the cause of compression which is focused on arterial compression, venous compression, or compression secondary to adhesions [12–16].

Thus, the purpose of this study was to determine the causes of trigeminal nerve compression in patients with trigeminal neuralgia undergoing microsurgical decompression, and compare the results with examination of the trigeminal nerve in patients without symptoms of trigeminal neuralgia undergoing microdissection for hemifacial spasm.

## Patients and methods

This prospective, case-controlled study included 99 patients with trigeminal neuralgia who were cured by microscopic decompression surgery at Yuquan Hospital of Tsinghua University from November 2008 to April 2010, and 101 patients with hemifacial spasm without any signs or symptoms of trigeminal neuralgia who were treated with microsurgical decompression in the same period and served as the control group. Patients with hemifacial spasm who were undergoing microdissection were chosen for the control group because the trigeminal nerve would be visible and could be examined during the course of surgery. No patient in the study had both trigeminal neuralgia and hemifacial spasm. This study was approved by the Institutional Review Board of the hospital, and all patients provided written informed consent.

All trigeminal neuralgia patients had failed or were unable to tolerate medical therapy, and some had undergone prior surgical procedures including gasserian ganglion block, nerve avulsion, retrogasserian rhizotomy, peripheral nerve block, and gamma knife surgery. Preoperative visual analogue scale (VAS) pain scores with 0 indicating no pain and 10 indicating the worst pain imaginable were obtained for trigeminal neuralgia patients. Microvascular dissection was performed in a standard manner [17]. Intraoperative findings recorded included the presence of compression and the vessel(s) causing the compression and the presence of adhesions. The relationship between the vessel and the nerve was categorized as either none, close to nerve, contact, or compression. All patients were followed up in the outpatient clinic.

Patients with hemifacial spasm also received standard microvascular dissection [18], and the trigeminal nerve was examined but not operated on. Observations of the trigeminal nerve were the same as those done for trigeminal neuralgia patients.

### Statistical analysis

Categorical data were presented as number with percentage and compared using Fisher’s exact test. Continuous data were presented as median with inter-quartile range (range from 25th to 75th percentiles) and compared using the Mann–Whitney test. Logistic regression model analysis was performed to find the independent impact factors of trigeminal neuralgia. Data were analyzed with using SPSS 15.0 statistics software (SPSS Inc., Chicago, IL, US), and a *p* < 0.05 was considered statistically significant.

## Results

A total of 99 patients (47 males and 52 females; median age: 57 years) with trigeminal neuralgia were included in the analysis. The course of trigeminal neuralgia ranged from 1 month to 30 years (median: 5 years). Eighty-five patients were treated with carbamazepine. The control group consisted of 101 patients (32 males and 69 females; median age: 47 years) with hemifacial spasm.

The characteristics of the patients in each disease group are summarized in Table 1. The patients with trigeminal neuralgia were significantly older than the patients with hemifacial spasm (median age: 57 vs. 47 years, *p* < 0.001); 70.7 % of the patients with trigeminal neuralgia were over 50 years old compared with only 41.6 % of the patients with hemifacial spasm (*p* < 0.001). More males were included in the trigeminal neuralgia group than in the hemifacial spasm group (47.5 vs. 31.7 %, *p* = 0.03).

**Table 1:** Summary for the patients characteristics by disease groups

	Trigeminal neuralgia (*n* = 99)	Hemifacial spasm (*n* = 101)	*p* value
Age (year)	57.0 (49.0, 64.0)	47.0 (40.0, 57.0)	<0.001*
Age group			
≤30	2 (2.0 %)	3 (3.0 %)	<0.001*
>30–≤50	27 (27.3 %)	56 (55.4 %)	
>50–≤70	59 (59.6 %)	39 (38.6 %)	
>70	11 (11.1 %)	3 (3.0 %)	
Gender			
Female	52 (52.5 %)	69 (68.3 %)	0.030*
Male	47 (47.5 %)	32 (31.7 %)	
Vein			
None	59 (59.6 %)	68 (67.3 %)	0.268
Close to nerve	5 (5.1 %)	9 (8.9 %)	
Contact	21 (21.2 %)	13 (12.9 %)	
Compression	14 (14.1 %)	11 (10.9 %)	
Artery			
None	17 (17.2 %)	58 (57.4 %)	<0.001*
Close to nerve	2 (2.0 %)	10 (9.9 %)	
Contact	12 (12.1 %)	25 (24.8 %)	
Compression	68 (68.7 %)	8 (7.9 %)	
Arachnoid adhesions			
Yes	3 (3.0 %)	18 (17.8 %)	<0.001*
No	96 (97.0 %)	83 (82.2 %)	
Course of the disease (year)	5.0 (2.0, 12.0)	NA	
VAS	7.0 (7.0, 9.0)	NA	

* Indicates a significant difference between disease groups

**Fig. 1:**
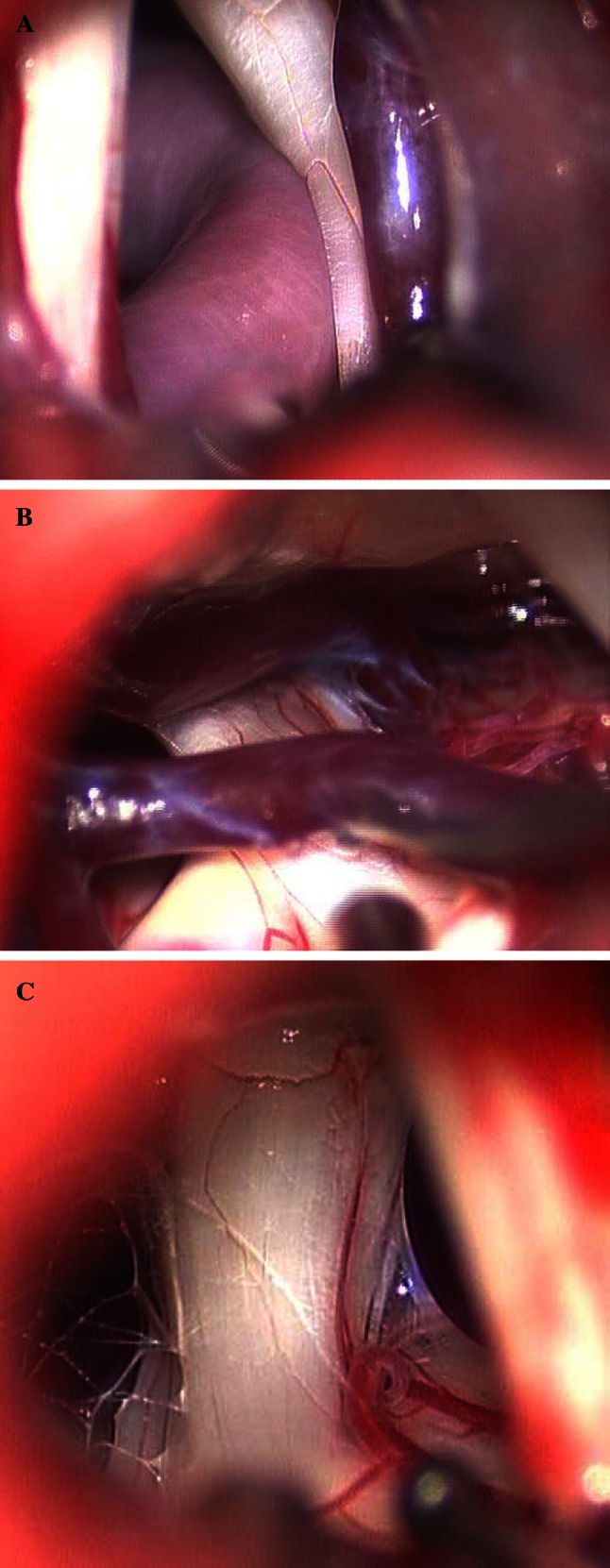
Representative intraoperative images of **a** artery compression and vein close, **b** vein compression, and **c** arachnoid adhesions

Representative images of artery compression, vein compression, and arachnoid adhesions are shown in Fig. 1. The relative position of an artery and the trigeminal nerve was significantly associated with trigeminal neuralgia (*p* < 0.001) (Table 1). Among the 200 patients, 76 had an artery compressing the trigeminal nerve, and 68 of these patients had trigeminal neuralgia (68.7 %), whereas only 8 had hemifacial spasm (7.9 %). Among 37 patients noted to have arterial contact with the trigeminal nerve, 12 had trigeminal neuralgia (12.1 %) and 25 had hemifacial spasm (24.8 %). Of 12 patients noted to have the artery close to but not touching the trigeminal nerve, only 2 had trigeminal neuralgia, whereas 10 had hemifacial spasm. Lastly, 21 patients were found to have arachnoid adhesions, and of these, 18 had hemifacial spasm while only 3 had trigeminal neuralgia (17.8 vs. 3.0 %, *p* < 0.001) (Table 1). There was no significant difference between the disease groups in the frequency of a vein compressing, being in contact, or being close to the nerve (Table 1).

The results of logistic regression analysis are shown in Table 2. After adjustment for age, gender, and other factors, patients with a vein compressing the nerve were more likely to have trigeminal neuralgia (OR = 5.21, *p* = 0.026) as were patients with a vein in contact with the nerve (OR = 4.22, *p* = 0.008). Patients with an artery compressing the nerve were more likely to have trigeminal neuralgia (OR = 42.54, *p* < 0.001). In addition, patients with arachnoid adhesions were less likely to have trigeminal neuralgia (OR = 0.15, *p* = 0.038).

**Table 2:** Summary for the impact factors of trigeminal neuralgia

	Crude OR (95 % CI)	*p* value	Adjusted OR (95 % CI)	*p* value
Age (year)	1.06 (1.03, 1.08)	<0.001*	1.07 (1.03, 1.11)	0.001*
Gender				
Male	1.95 (1.10, 3.47)	0.023*	1.37 (0.60, 3.08)	0.453
Female	Reference		Reference	
Vein				
None	Reference		Reference	
Close to nerve	0.64 (0.20, 2.02)	0.446	2.69 (0.61, 12.00)	0.193
Contact	1.86 (0.86, 4.04)	0.116	4.22 (1.46, 12.22)	0.008*
Compression	1.47 (0.62, 3.48)	0.384	5.21 (1.22, 22.15)	0.026*
Artery				
None	Reference		Reference	
Close to nerve	0.68 (0.14, 3.42)	0.642	0.87 (0.13, 5.73)	0.883
Contact	1.64 (0.68, 3.93)	0.269	1.69 (0.56, 5.12)	0.351
Compression	29.00 (11.67, 72.08)	<0.001*	42.54 (13.39, 135.19)	<0.001*
Arachnoid adhesions				
Yes	0.14 (0.04, 0.51)	0.003*	0.15 (0.03, 0.90)	0.038*
No	Reference		Reference	

* Indicates a significant association to trigeminal neuralgia

Lesions were frequently found at the maxillary branch, and most of them involved one nerve. All trigeminal neuralgia patients were followed up for a minimum of 2 years. Patients report no improvement in their symptoms had been excluded from this study.So all of these 99 patients in analysis were cured after surgery.

## Discussion

The findings of this study indicate that compression of the trigeminal nerve by an artery is most often the cause of trigeminal neuralgia rather than compression of the nerve by a vein. These results indicate that in patients with arterial compression, complete decompression is required and removal of adhesions are also necessary, but no treatment of the vein is needed. This study is unique in that the control group consisted of patients without symptoms of trigeminal neuralgia in whom the trigeminal nerve could still be examined. Prior studies used cadavers as the control group which may have affected the findings. During microvascular decompression in patients with hemifacial spasm, the arachnoid above the auditory nerve is opened and the trigeminal nerve can be observed.

Much of the theoretical evidence for the treatment of trigeminal neuralgia with microvascular dissection is from cadaveric anatomic studies [19]. However, anatomic changes noted in cadavers do not necessarily reflect anatomic variations seen intraoperatively. Few animal models have been developed to study compression of the trigeminal nerve root [20]. Advances in imaging techniques have allowed for detailed examination of neurovascular structures and studies in patients with trigeminal neuralgia have demonstrated contact between vessels and the trigeminal nerve [21–24]. The majority of the evidence for vascular compression of the trigeminal nerve as the cause of trigeminal neuralgia is from studies examining outcomes after microsurgical decompression [12–15, 17].

Zhang et al. [15] recently reported the results of 154 consecutive patients with trigeminal neuralgia who underwent microvascular decompression, and the initial and 5-year pain-free rates were 84 and 72 %, respectively. Obvious vessel compression observed during surgery was significantly associated with long-term pain relief (OR = 3.2). Sekula et al. [12] reported that 31 of 36 patients (86 %) with a mean age of 73 years had excellent outcomes after microvascular decompression for trigeminal neuralgia. Tucer et al. [13] reported good outcomes in 37 patients who received microvascular decompression for trigeminal neuralgia, and found that the only factors affecting prognosis of the surgery were intraoperative detection of compression and signs of compression on preoperative magnetic resonance imaging (MRI) and MR angiography (MRA) studies. Oesman and Mooij [14] also reported good results of microvascular decompression, and that immediate resolution of symptoms postoperatively was the most important predictor of long-term relief of pain. Despite the encouraging results of individual studies, a recent Cochrane systematic review by Zakrzewska and Coakham [25] found that although microvascular decompression is likely the most effective therapy in correctly diagnosed patients, high-quality prospective studies are lacking.

Vein compression is frequently found during surgery in patients with trigeminal neuralgia, but in many cases the site of vein compression is not consistent with the area of pain, and decompression of the vein by moving the vessel of ligation with electrocoagulation does not resolve the symptoms or only transient improvement is observed. Few studies, have reported trigeminal neuralgia due to venous compression alone [16]. During microvascular dissection, manipulation of the veins is associated with a risk of rupture and ligation of the veins may result in the obstruction of venous return in the brainstem and cerebellum, or possible congestive cerebral infarction. Our finding that trigeminal neuralgia was not associated with venous compression indicates that there might be no requirement for special treatment of the veins, which may reduce the risk for venous injury and venous return obstruction. Since most arteries are tortuous, and their stiffness increases with age, this increasing stiffness can result in compression of the root of the trigeminal nerve, leading to demyelination and subsequent trigeminal neuralgia. On the contrary, veins do not bear high blood pressure, their walls are thin, and their location is relatively fixed, and thus, veins tend not to become stiff and/or tortuous with age and are therefore not likely to cause compression of the nerve.

## Conclusions

The findings of this study indicate that compression of the trigeminal nerve from veins was not a cause of trigeminal neuralgia. These results indicate that while in patients with arterial compression of nerve, complete decompression is required and removal of adhesions is also necessary, treatment of the vein may not need to be a priority when performing microvascular dissection.
